# New perspectives on the role of vitamins in bipolar disorders: are there any relationships with outcomes?

**DOI:** 10.1192/j.eurpsy.2023.465

**Published:** 2023-07-19

**Authors:** G. De Iorio, D. Marazziti, L. Massa, M. Violi, M. G. Carbone, A. Arone, S. Palermo, W. Flamini, L. Massoni, L. Dell’Osso

**Affiliations:** ^1^Clinical and Experimental Medicine, University of Pisa, Pisa; ^2^Saint Camillus International University of Health and Medical Sciences – UniCamillus, Rome; ^3^Medicine and Surgery, University of Insubria, Varese, Italy

## Abstract

**Introduction:**

Vitamin B12, folic acid and homocysteine play a key role in cellular functioning as part of “one-carbon metabolism”, a biochemical pathway involved in many essential biological processes, such as DNA synthesis. Therefore, imbalance involving these micronutrients might impair neurological functioning as well. Vitamin B12 has been implicated in the onset of a wide range of neuropsychiatric symptoms/disorders, like mood disorders, anxiety, hallucinations and delirium. Altered levels have been reported in mood disorders (MDs), but available literature particularly focuses on major depression (MDD), while the information in bipolar disorders (BDs) is still limited.

**Objectives:**

The present study aimed at assessing vitamin B12, homocysteine and folic acid in bipolar inpatients and detecting any relationship with clinical features or outcome measures.

**Methods:**

A total sample of 69 inpatients was selected. Diagnoses of bipolar disorder I (BDI), II (BDII), schizoaffective disorders, and MDD, were assessed according to DSM-5 criteria. The Mini International Neuropsychiatric Interview (MINI), Hamilton Rating Scale for Depression (HRSD), Young Mania Rating Scale (YMRS) and Clinical Global Impression-Severity (CGI) scales were used to complete the psychopathological evaluation. The blood parameters were measured according to common clinical-chemical methods.

**Results:**

About 50 % of bipolar patients (34) showed significantly lower vitamin B12, and 14 higher homocysteine levels than normative values. No differences were noted between genders, except for a slightly higher rate of women showing lower homocysteine, phase of illness, intake of psychotropic drugs, or dietary habits. Folic acid levels were normal in most of the sample. Patients with a family history of suicide showed significantly lower levels of vitamin B12.

**Conclusions:**

These results suggest that implementing the assessment of vitamin B12, homocysteine and folic acid in patients with BD in routine clinical practice could be a useful as well as simple, non-invasive and cheap tool. Although other studies are necessary, the present findings that lower levels of vitamin B12 seem typical of patients with a family history of suicide independently from the phase of illness, suggests that they might constitute a possible predictor of this tragic outcome.

**Disclosure of Interest:**

None Declared

